# Determining Airborne Concentrations of Spatial Repellent Chemicals in Mosquito Behavior Assay Systems

**DOI:** 10.1371/journal.pone.0071884

**Published:** 2013-08-28

**Authors:** Nicholas J. Martin, Philip A. Smith, Nicole L. Achee, Gerald T. DeLong

**Affiliations:** 1 Viral and Rickettsial Diseases Department, U. S. Naval Medical Research Center, Silver Spring, Maryland, United States of America; 2 Preventive Medicine and Biometrics Department, Uniformed Services University of the Health Sciences, Bethesda, Maryland, United States of America; 3 Health Response Team, U. S. Department of Labor – Occupational Safety and Health Administration, Sandy, Utah, United States of America; 4 U. S. Naval Inspector General, Portsmouth, Virginia, United States of America; University of Crete, Greece

## Abstract

**Background:**

Mosquito behavior assays have been used to evaluate the efficacy of vector control interventions to include spatial repellents (SR). Current analytical methods are not optimized to determine short duration concentrations of SR active ingredients (AI) in air spaces during entomological evaluations. The aim of this study was to expand on our previous research to further validate a novel air sampling method to detect and quantitate airborne concentrations of a SR under laboratory and field conditions.

**Methodology/Principal Findings:**

A thermal desorption (TD) gas chromatography-mass spectrometry (GC-MS) method was used to determine the amount of dichlorodiphenyltrichloroethane (DDT) in samples of air. During laboratory experiments, 1 L volumes of air were collected over 10 min intervals from a three-chamber mosquito behavior assay system. Significantly higher levels of airborne DDT were measured in the chamber containing textiles treated with DDT compared to chambers free of AI. In the field, 57 samples of air were collected from experimental huts with and without DDT for onsite analysis. Airborne DDT was detected in samples collected from treated huts. The mean DDT air concentrations in these two huts over a period of four days with variable ambient temperature were 0.74 µg/m^3^ (n = 17; SD = 0.45) and 1.42 µg/m^3^ (n = 30; SD = 0.96).

**Conclusions/Significance:**

The results from laboratory experiments confirmed that significantly different DDT exposure conditions existed in the three-chamber system establishing a chemical gradient to evaluate mosquito deterrency. The TD GC-MS method addresses a need to measure short-term (<1 h) SR concentrations in small volume (<100 L) samples of air and should be considered for standard evaluation of airborne AI levels in mosquito behavior assay systems. Future studies include the use of TD GC-MS to measure other semi-volatile vector control compounds.

## Introduction

Mosquitoes are capable of transmitting numerous diseases including malaria, dengue fever, yellow fever, Japanese encephalitis, and West Nile fever among others [1], [2]. Due to the geographic distribution of mosquitoes, as many as three billion people are at risk of infection with at least one mosquito-borne disease [3], [4]. Of those at risk, malaria causes the highest burden of disease with an estimated 216 million cases and 655,000 deaths reported in 2012 [3]. In addition, infection with one of the four serotypes of dengue virus is responsible for up to 400 million infections annually [5], with up to 500,000 cases progressing to the life-threatening dengue hemorrhagic fever [4].

Two of the primary strategies to control mosquito-borne diseases as recommended by the World Health Organization (WHO) are the use of long-lasting insecticide-treated nets (LLINs) and indoor residual spraying (IRS) to reduce exposure to mosquitoes [6], [7]. However, only twelve compounds in four chemical classes are currently available for LLINs and IRS [7]. In an effort to identify new active ingredients (AI) and/or innovative chemical paradigms of vector control, such as the use of spatial repellents (SR) to modify mosquito behavior [8], [9], entomological assays have been developed to describe specific vector response following exposure to an AI [10]–[12]. These include both laboratory and field test systems that measure repellency (deterrence or reduction in mosquito entry), irritancy (increased exit), and mortality [10], [13]–[16]. Dichlorodiphenyltrichloroethane (DDT), a compound approved by the WHO for use in IRS operations, has been the focus of anopheline behavioral evaluations. In subsequent studies, SR activity of DDT has also been evaluated against both male and female *A. aegypti* mosquitoes [17], [18]. Combined, these studies demonstrate that DDT elicits SR activity in mosquito vectors [19].

At the time the studies mentioned previously were conducted, there were no published analytical methods to measure the concentration of airborne DDT over short sampling intervals (≤1.0 h); therefore, the concentration of DDT relevant to SR activity in test systems could not be determined with temporal resolution. Although defining the short-duration concentration of airborne DDT was not a specific objective of previous evaluations, it is now recognized as a critical component in the development of novel or reformulated vector control compounds. This is because an understanding of the specific conditions required to generate sufficient airborne concentrations of a SR chemical to repel mosquitoes will allow identification of operationally significant parameters relevant to SR control strategies. These parameters include product format, placement in a given space (e.g., home), required AI loading levels to elicit minimum thresholds of mosquito responses, effective distance, and environmental conditions such as temperature, humidity, and wind speed, that may affect airborne SR concentrations.

Here we report on a thermal desorption (TD) gas chromatography-mass spectrometry (GC-MS) method, previously developed in our laboratory [20], to determine the concentrations of airborne DDT in samples of air collected from laboratory and field mosquito behavior assay systems. Specific objectives of this study included: 1) validating a difference in airborne DDT concentrations from spaces with and without DDT treatment and 2) describing the role of the TD GC-MS method to measure concentrations of airborne AI.

## Experimental Methods

### Ethics Statement

Permission was obtained from the Thailand Armed Forces Development Command prior to conducting field evaluation in Pu Teuy Village, Sai Yok District, Kachanaburi Province, Thailand (14u209110N, 98u599450E).

### Materials

Analytical standards (≥99% purity) for 2, 4′ and 4, 4′ isomers of dichlorodiphenyldichloroethane (DDD), dichlorodiphenyldichloroethylene (DDE), and DDT were obtained from Accustandards (New Haven, CT). Stock solutions were prepared in pesticide-free isooctane (Honeywell Burdick and Jackson, Morristown, NJ) for laboratory experiments or reagent grade acetone (Fischer Scientific, Pittsburgh, PA) for field experiments. Stock solutions were stored in the dark at 4°C until testing. Ultra high purity (UHP) He and N_2_, acquired from local suppliers, were used for carrier gas and TD system cold trap dry purge gas respectively, during laboratory (Air Gas, Bethesda, MD) and field (Air Gas, Bangkok, Thailand) experiments.

### Analytical Methods

A TD GC-MS method, previously developed in our lab [20], was used for near real-time analysis of laboratory and field samples. For field analyses, the TD GC-MS instrument and supporting equipment items were shipped to Bangkok, Thailand and later transported to the experimental hut site of Pu Tuey Village. The TD GC-MS instrument was operational within 24 h of transportation to the field site.

### Sample Introduction

A Unity 2 thermal desorber (Markes International, Ilantrisant, UK) was connected by a heated transfer line (200°C) to an Agilent 5975T GC-MS instrument (Santa Clara, CA) with a low thermal mass (LTM) column assembly. The transfer line was connected directly to the analytical column through the heated injector body with the liner removed.

Laboratory calibration curves were generated by quantitatively loading 1.0 µl of diluted stock solution (1.0–250.0 ng DDT in 1.0 µL isooctane) into a sampling tube. Control samples (sample tubes spiked with known amounts of DDT) were analyzed every 10–20 samples. Experimental samples were not analyzed if controls were not within ±15% of expected values. Standards of DDT in isooctane were prepared in Bethesda, MD and were packaged according to international shipping requirements for transport at ambient temperature to Thailand for field calibration of the TD GC-MS system.

A two-stage split TD method was used with 75 mL/min flow through the tube during desorption at 300°C (10 min) onto a low volume focusing trap. The trap was maintained at 20°C during primary tube desorption with 15 mL/min He flow through the trap and a 60 mL/min flow to the split vent. The trap was then ballistically heated to 300°C and the focused analytes were transferred onto the GC column without split (10 min), providing an overall split ratio of 5∶1 for this TD method.

### GC-MS Analysis

A DB-1 open tubular fused silica analytical column was used (J & W Scientific, Folsom, CA; 30 m×0.25 mm i.d.×0.25 µm film thickness), with helium used as the desorption and carrier gas for separation completed at constant pressure (12 PSI). The transfer lines from the heated injector body to the resistively heated LTM GC column and from the small convection oven to the MS detector were maintained at 250°C and 280°C, respectively. The initial GC column temperature was held at 50°C for 30 s, followed by a 50°C/min ramp to 200°C (no hold), 10°C/min ramp to 270°C (no hold), and 30°C/min to 300°C (held for 30 s).

Electron ionization (70 eV) was used with a 2.75 min solvent delay. Selected ion monitoring (SIM)/scan mode was used, scanning *m*/*z* 75 to 360 at 3.75 scans/s, providing at least 10 scans across the relevant GC peaks. Quantitation was performed using *m*/*z* 165 and 235 SIM data.

### Sample Collection

Samples of air were collected using tubes (89 mm×4 mm i.d.×6.4 mm o.d.) packed with 200 mg of Tenax-TA adsorbent (Markes International Ilantrisant, UK). Tubes were conditioned at 300°C for 20 min with a constant N_2_ stream (30 mL/min) prior to use. Low-flow personal air sampling pumps (Model 222, SKC Inc., Eighty Four, PA) were set to operate with a flow rate of 100 or 200 mL/min in the laboratory and field, respectively. Pumps were calibrated before and after sampling using a device to measure volumetric flow rate (Defender 510, Bios International, Butler, NJ).

### Time-delayed Analysis

During field experiments, samples were analyzed following variable delays post-collection. To assess the impact of time-delayed analysis on DDT recovery, replicate TD tube samples (n = 4) were prepared by spiking 100 ng DDT in 1.0 µl isooctane onto the metal screen of a TD tube at the sampling inlet. Analysis by TD GC-MS was conducted immediately, and after delays of one or three days. Before analysis, sampling tubes were sealed with brass caps and polytetrafluoroethylene ferrules and stored at 4°C.

### Laboratory Sample Collection

**Figure 1:**
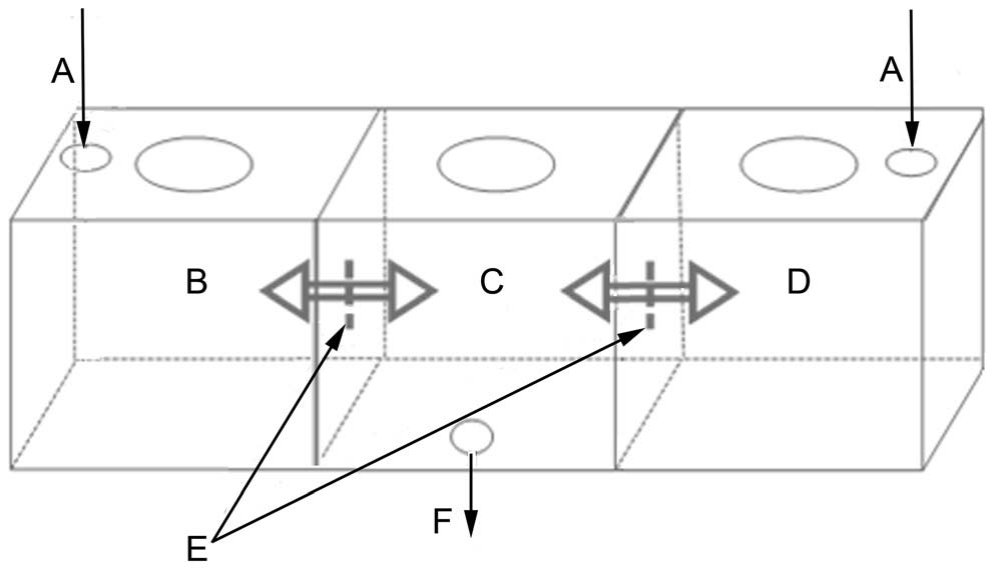
A schematic diagram of the three chamber system used to study mosquito behavior. Each chamber was 30.5 cm×30.5 cm×30.5 cm (28.4 L) with a 10 cm hole cut into a removable acrylic lid. A: lab air supply (5 L/min) measured with a rotameter, B: metal treatment chamber, C: acrylic mosquito introduction chamber, D: metal control chamber, E: closable funnels opened during exposures studies to allow mosquitoes, air flow, and airborne chemical to move between the chambers, F: vacuum exhaust (10 L/min).

A three-chamber mosquito behavior assay system was used for laboratory evaluations (Fig. 1) [21]. The three chambers represented: treatment (containing DDT-treated textile); central (point of mosquito introduction); and control (containing DDT-free fabric). Each chamber was a 28.4 L cube with a 10 cm hole cut into a removable clear acrylic lid. The treatment and control chambers were constructed from metal with acrylic lids and were fitted with beveled funnels that allow passage of mosquitoes originating from the central chamber during tests. The central chamber was made entirely of clear acrylic.

Chemical treatment was matched to standard mosquito behavior evaluation protocols. White polyester (mesh size 24×20/inch; Bioquip Products, Rancho Dominquez, CA) or nylon (No 4-2; G Street Fabrics, Rockville, MD) textile was treated with DDT solution at 0.09–2.0 g/m^2^, corresponding to 0.4–100% of the WHO recommended IRS loading rate [6] using acetone and isooctane diluents as described previously [17]. Textile was prepared to cover 100%, 75%, 50% or 25% surface area of treatment and control chambers. Control fabric was prepared with solvent only. Fabric panels were treated 30–60 min prior to starting an assay and allowed to air-dry on a drying rack for 15–30 min before placement in the test system. The material remained in the treatment or control chambers for the duration of a test day.

Filtered air (5.0 L/min) measured with a rotameter (RMA-5-BV Flowmeter, Dwyer Instruments, Inc., Michigan City, IN) for each inlet was supplied to the assay system through two inlets; one each in the treatment and control chambers. The 10 L/min of supplied air was exhausted from the system through the mosquito introduction chamber. Before air sampling was performed each chamber was filled with argon and a hand-held thermal conductivity detector was passed along the surfaces of the chamber joints to determine if the welded and sealed joints were airtight.

Airflow velocity was measured at 27 points inside each chamber to determine if differences in airflow existed within and between the chambers using an anemometer (VelociCalc 9555, Thermo Scientific Inc., Shoreview, MN). Air changes per hour in the treatment chamber were determined by introducing a high concentration of CO_2_ into the chamber and then measuring the decay of this gas with a portable meter equipped with a non-dispersive infrared absorbance detector (MultiRAE IR, RAE Systems, San Jose, CA) [22].

The sampling pumps were kept outside of the test chambers during sample collection and connected to the sample tubes by inert tubing (R3606 tubing; Saint-Gobain Performance Plastics, Aurora, OH). Pump flow rate (100 mL/min) was checked daily, both before and after sample collection with a sampling tube inline, to verify sampling rate was within ±5% of the set value. The average pump flow rate and sample collection time were used to calculate the volume of air sampled. The temperature and relative humidity of the testing room (recorded at the start of each day) were 26°C–31°C and 10%–20%, respectively.

### Field Sample Collection

Air samples were collected from inside experimental huts used for mosquito behavior evaluations [23]. The construction and design of the experimental huts has been previously described [10]. Briefly, huts were 4.0 m wide, 5.0 m deep and 2.5 m tall, with three windows (1.1 m×1.2 m) and one door (0.8 m×2.0 m) comprising a total internal volume of 50 m^3^. Chemical treatment matched laboratory evaluations. Polyester fabric (19.8 m^2^ total per hut) that corresponded to 50% of the interior wall surface area was treated with 2.0 g/m^2^ of DDT dissolved in acetone one day prior to placement in treatment huts (huts B and C). Polyester textile treated with acetone only was positioned inside the control hut (hut A).

**Figure 2:**
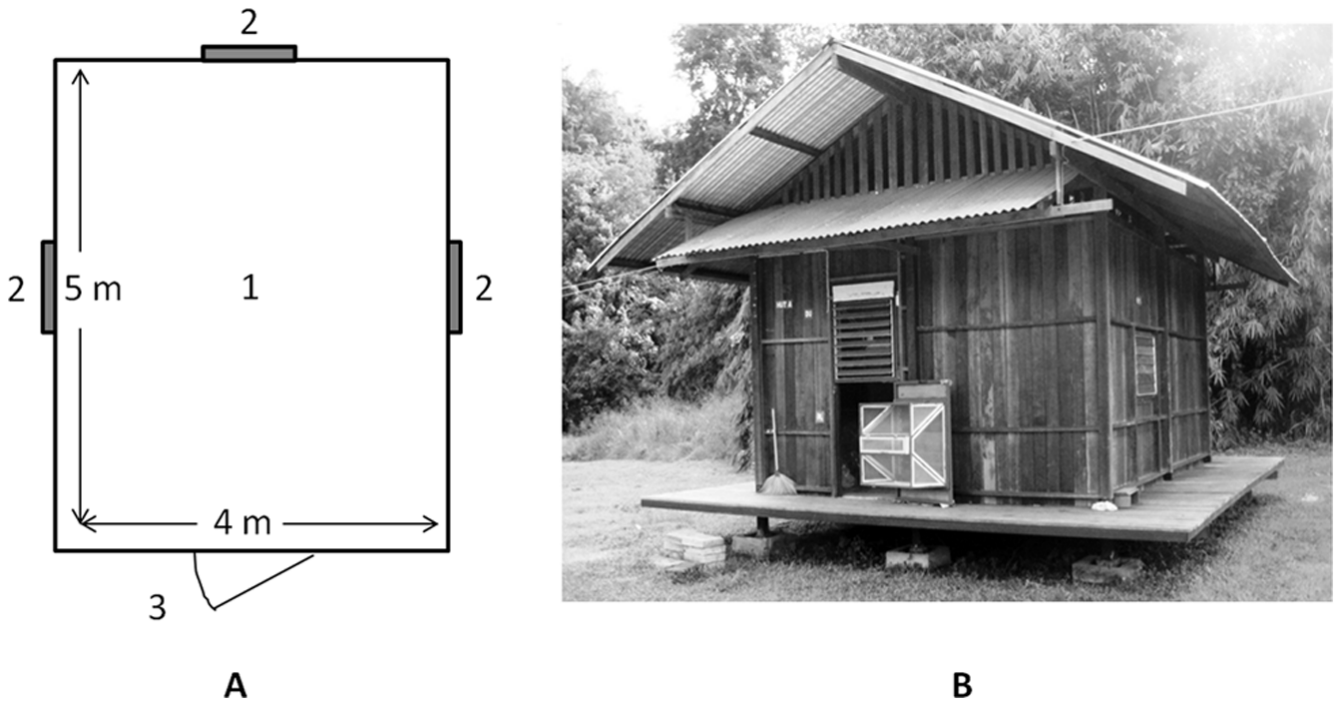
Diagram (A) and picture (B) of experimental huts. The sampling pumps were placed on 1.5 m tall stands in the approximate center of each hut (#1 panel A). Each hut had three screened windows (#2 panel A) and one screened door (#3 panel A) allowing air into the hut from the outside.

In the field, samples of air were collected during 60 min intervals inside the three experimental huts (Fig. 2). The flow rate of the sampling pumps was measured through a representative sample collection tube at 200 mL/min (±2 mL/min) before and after sample collection. The average pump flow rate and sample collection time were used to calculate the volume of air sampled. Pumps were mounted on wooden stands in the center of each hut approximately 1.5 m above the floor of the hut. Samples were collected over 1 h intervals between 0600–1800 during 9–12 October 2010. Outdoor temperature and wind speed were measured outdoors at a location central to the huts while the temperature was measured continuously inside each hut. The indoor air change rate was determined using the decay of CO_2_ by the same portable gas meter used to make similar determinations in the three-chamber laboratory system.

### Statistical Analysis

Statistical analyses were completed in Sigma Plot for Windows (Version 11.0, Systat Software, Chicago, IL). Analysis of variance (ANOVA) was used to assess the impact of delayed analysis on the mass of DDT remaining in spiked sampling tubes immediately, and after delays of one and three days. For laboratory evaluations, the inter-day and inter-chamber variations in the concentration of airborne DDT were evaluated by ANOVA, comparing the concentrations measured in the treatment chamber on different testing days and in different chambers, respectively. Differences in the airflow rate measured in each chamber of the laboratory mosquito behavior assay were compared by ANOVA. Holmes-Sidak (parametric) and Tukey (non-parametric) *post-hoc* tests were performed for all analyses (as appropriate). A p value of less than 0.05 indicated statistical significance for all analyses.

## Results

### Laboratory Sampling

Argon leakage was detected at each non-welded seal indicating the box model system was not airtight and that air could be supplied or removed from the system independent of the inlets and exhaust. The median air velocity was 1.0 cm/s in each chamber of the laboratory system. A Kruskal-Wallace one-way ANOVA test did not demonstrate a significant difference between chambers (H = 1.104; p = 0.576). Higher air velocities (2.5–21.8 cm/s) were measured directly below the inlets in the treatment (Fig. 1B) and control (Fig. 1D) chambers.

**Figure 3:**
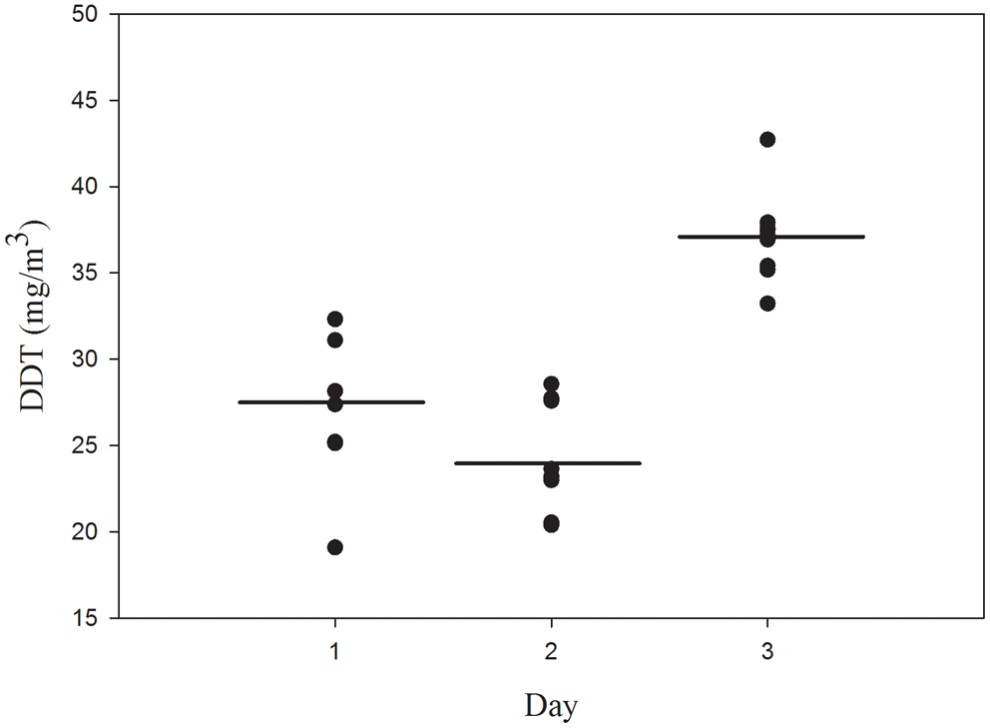
Scatter plot of DDT air concentration in samples collected on three separate days from the treatment chamber of the three chamber system. Polyester fabric treated with 0.9 g/m^2^ 4, 4′ DDT was prepared each day and placed on 100% of the wall surface area of the treatment chamber. The mean airborne DDT concentration (denoted by a solid line for each day) was significantly different between days (one way ANOVA; F = 33.664; P<0.001). The DDT air concentration measured on Day 3 was significantly higher) than the levels measured on Days 1 and 2 (Holm-Sidak *post hoc*; p<0.001 for both comparisons). The DDT air concentration measured on Day 1 was significantly higher than the levels measured on Day 2 (Holm-Sidak *post hoc*; p = 0.041).

A total of nine samples of air (1.0 L) were collected during 10 min sampling periods over a three day period to assess the stability of airborne DDT concentrations in the chamber containing DDT treatment (100% coverage at 0.09 g/m^2^) (Fig. 1B). The intra-day variation was assessed by calculating the daily relative standard deviation (RSD). The RSD was 16.3%, 13.8%, and 7.0% for days 1–3, respectively. Inter-day variance was assessed to determine the effect of sample preparation (polyester independently prepared each day before placement in the test chamber) and time (Fig. 3). Results showed a significant difference between the mean DDT air concentrations measured on each of the three days (F = 33.664; p<0.001). The DDT air concentration measured on Day 3 was significantly higher than levels measured on Days 1 and 2 (Holm-Sidak *post hoc*; p<0.001).

Examination of chamber-specific DDT air concentrations using 25% coverage at 2 g/m^2^ indicated large intra-chamber variation (>100%; n = 9). The intra-chamber variation calculated for each chamber was 110%, 139% and 197% for treatment, central and control, respectively. This may be due in part to the percentage of samples below the limits of quantitation (treatment: 11.1%, central: 37.0%, and control: 18.5%) and detection (treatment: 7.4%, central: 55.6%, and control: 63.0%). Samples between the limit of quantitation and detection were assigned the value of half the limit of quantitation (0.5 ng) and samples without detectable levels of DDT were assigned the value 0 ng.

**Figure 4:**
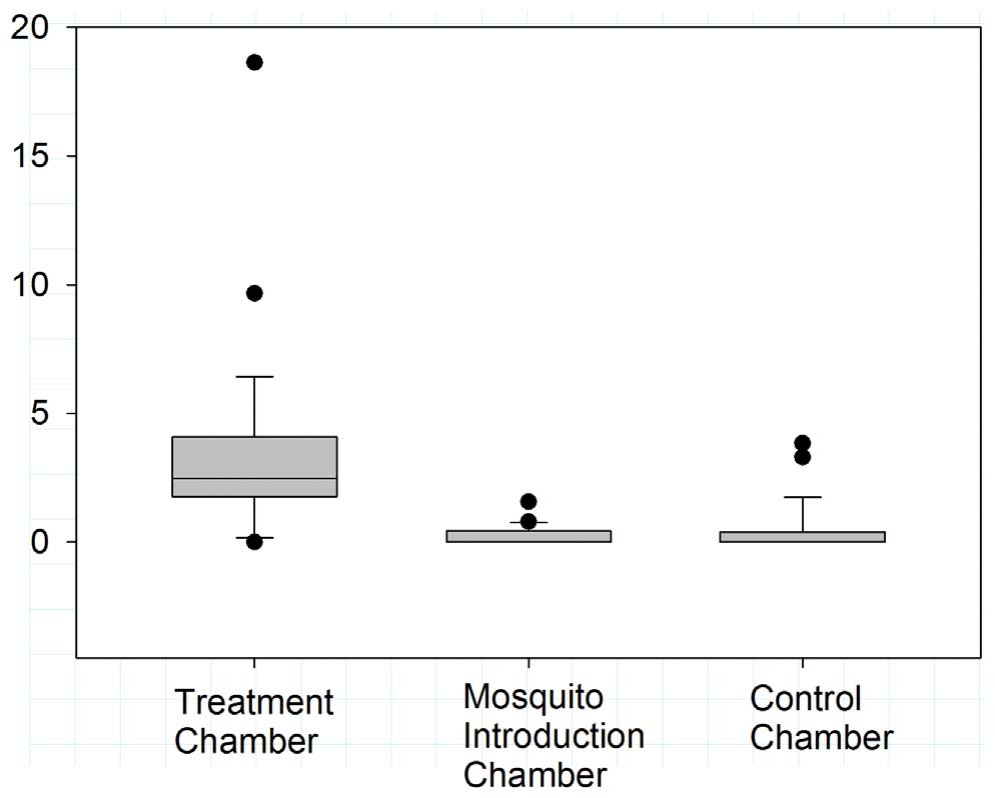
Box-and-whisker plot of DDT air concentration in samples collected from the treatment (Fig. 1 B), mosquito introduction (Fig. 1 C), and control (Fig. 1 D) chambers of the laboratory system (black circles denote samples above or below the 90% and 10% percentiles, respectively). Nylon fabric treated with 0.09 g/m^2^ 4, 4′ DDT was prepared each day and placed on 50% of the wall surface area of the treatment chamber. The median airborne DDT concentration was significantly different between days (Kruskal-Wallis one-way ANOVA; H = 35.461; P<0.001). The DDT air concentration measured for the treatment chamber was significantly higher than the levels measured in the mosquito introduction and control chambers (Tukey *post hoc*; p<0.05 for both comparisons).

The median concentration of airborne DDT was not significantly different in the treatment chamber between the three days (Kruskal-Wallis ANOVA; H = 5.190; p = 0.075) indicating that a similar concentration of airborne DDT was generated during the three-day experiment. However, the median concentration of airborne DDT was significantly different between the three chambers (Fig. 4; Kruskal-Wallis ANOVA; H = 35.461; p<0.001) with median concentration significantly higher in the treatment chamber compared to the central and control chambers (Tukey *post hoc*; p<0.05).

### Field Sampling

The results of field analyses are summarized in Table 1. The TD method produced a linear GC-MS response (R^2^ = 0.933) from TD tubes spiked in the field with DDT (5.0 to 100.0 ng). Relative standard deviations were 49.7, 26.0, 18.8, 15.1 and 24.3 for the 5 ng, 10 ng, 20 ng, 50 ng and 100 ng calibration points, respectively. Method performance in the field did not match that performance obtained in the laboratory with respect to linearity, precision, and sensitivity [20]. To account for this, the variance_DDT Predicted_ was calculated for 10 and 50 ng loading values [24]. The mean variance_DDT Predicted_ was ±4.895 ng (10 ng: ±4.38 ng; 50 ng: ±5.41 ng) resulting in a variance_DDT Predicted_ for calculated DDT air concentrations of ±0.41 µg DDT/m^3^ air. A total cycle time of 25 min per sample allowed a sample throughput of approximately two samples per hour in the field. This relatively short analysis time (compared to ∼18 h with conventional solvent extraction) facilitated completion of near real-time DDT detection and quantitation. Analyses of the control (n = 18) and hut (n = 57) samples were completed in approximately 40 h.

**Table 1:** Mean DDT air concentrations, with standard deviation in parentheses, determined in the control hut (A) and two treatment huts (B and C).

Hut	Samples Collected	Temperature (°C)	Relative Humidity (%)	[DDT]_air_ (µg/m^3^)	[DDT] ± variance_DDT Predicted_ (µg/m^3^)	Percent quantifiable samples (n)
**A**	10	25.9±3.3	85.2±11.6	ND*	ND*	10% (1)*
**B**	17	25.7±3.3	83.5±11.7	0.74±0.45	0.33–1.15	64.7% (11)
**C**	30	25.8±3.0	85.6±13.2	1.42±0.96	1.01–1.83	93.3% (28)

* One sample analyzed with 1.22 µg/m^3^ DDT; a labeling error is suspected.

**Figure 5:**
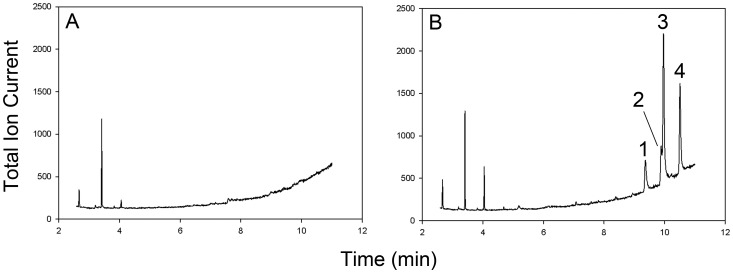
Selected ion (m/z 165 and 235) chromatograms for field control hut (A) and treatment hut (B). 4, 4′ DDE (peak 1), 4, 4′ DDD (peak 2), and 2, 4′ DDT (peak 3) were detected with the target analyte 4, 4′ DDT (peak 4). Peak identity was confirmed by retention time and mass spectral data from analytical standards.

Fifty-seven samples of air were collected with TD tubes from the three experimental huts (Fig. 2). Overall, the amount of airborne DDT measured in samples of air collected during the four days at the field site ranged from non-detectable to 4.30 µg/m^3^ (Table 1). DDT detection occurred in 83% of samples from treated huts (huts B and C) and in one sample from the control hut (labeling error suspected) as previously reported [8]. While quantitation of airborne DDT concentration was completed by measuring the area under the curve for SIM analysis of 4, 4′ DDT (Fig. 5B; peak 4), three other DDT-related GC peaks were also noted. The earlier eluting peaks are likely DDT degradation products 2, 4′ DDD (Fig. 5B peak 1), 4, 4′ DDD (Fig. 5B peak 2), and the DDT isomer 2, 4′ DDT (Fig. 5B peak 3) based on elution order and corresponding full scan mass spectra [20]. The mean indoor air temperature measured in each hut (Table 1) did not show a statistically significant difference between huts. The air change rate measured in hut C was approximately six changes per hour (∼300,000 L/hr) based on tracer gas decay measurement, with replacement air supplied by the three windows, one door, and through the walls (determined by visual smoke test). The air volume collected during each sampling interval (∼12 L) represented ∼0.004% of the total volume present in the field system.

Time-delay analysis experiments indicated the mean recovery from sampling tubes spiked with DDT was 94.4 ng (n = 4; SD = 6.6 ng), 89.6 ng (n = 4; SD = 3.3 ng), and 86.1 ng (n = 4; SD = 6.1 ng) for samples analyzed immediately, after one day (mean delay 23.12 h), and after three days (mean delay 72.96 h), respectively. A one-way ANOVA did not demonstrate a significant difference between groups (*F* = 2.279; *p* = 0.158). Additionally, the DDT recovery following one and three days delayed analysis were acceptable (±15% of the starting DDT mass) as defined by the U.S. Environmental Protection Agency (EPA) for analysis of control samples [25]. This suggests that delays of up to three days between sample collection and analysis did not impact DDT recovery from TD sampling tubes.

## Discussion

Quantifying the concentrations of airborne SR chemicals during laboratory and field mosquito behavior studies is critical to understanding the relationship between chemical exposure and mosquito behavior [26]. Such information can be used, in part, to establish entomological correlates of health outcomes related to human protection such as percent reduction in mosquito entry into a treated space, or biting rates. This report describes important performance details for a TD GC-MS analytical method introduced previously [26], to quantify concentrations of airborne DDT in both laboratory and field mosquito assay systems.

Standard environmental sampling methods were not designed to measure airborne AI in samples collected during 10–60 min intervals used in the mosquito behavior assays evaluated in this report. Additionally, these methods rely on solvent extraction to remove compounds of interest from the sample media prior to analysis, reducing the method sensitivity and increasing the analytical method complexity. The TD GC-MS method developed previously [20] and described in detail in this report addresses the limitations of the standard methods with respect to sampling duration with a simplified sample introduction method. All sample preparation was eliminated with the TD GC-MS method as metal tubes packed with sampling media were inserted directly into the TD unit following sampling collection reducing method complexity and analysis time. Sample recovery was also improved compared to traditional methods; we previously reported >90% sample recovery [20] compared to <1% possible with solvent extraction. The TD GC-MS method was sensitive enough to measure airborne DDT samples of air collected during 10 and 60 min intervals collected from the mosquito behavior systems in the laboratory and field, respectively. Collection of large volumes of air from mosquito behavior systems could have unintended impact on the behavior of AI and mosquitoes within the system. In the field system approximately 0.004% of the total air volume was sampled during each sampling interval, reducing the impact on the system dynamics with respect to air change rate, AI emission rate and chemical movement.

Longer duration samples provide information regarding the time-weighted average airborne AI concentrations, but cannot provide temporal resolution of high and low concentration values that occur throughout the sampling interval. Efforts to understand mosquito behavior following exposure to SR must include measurement of airborne AI concentrations over brief periods of time to ensure that excursions above and below an average concentration can be identified and correlated with altered insect behavior. The use of sorbent sampling with TD-based analyte introduction provides a substantial improvement for sampling a dynamic field system in which AI concentrations are expected to fluctuate due to uncontrolled environmental conditions. A short-duration TD method is also important for measuring AI concentration fluctuations in laboratory systems assumed to be stable.

During the assessment of the laboratory assay system, significantly higher concentrations of airborne DDT were observed within the treatment chamber compared to the central and control chambers. This finding validated the assumption that mosquitoes placed in the introduction chamber (central chamber) would be exposed to airborne DDT and that a gradient exists between the treatment (highest concentration) and the control (lowest concentration) chambers. This finding supports reports on the SR action of DDT by other investigators in which mosquitoes exposed to DDT-treated materials, but not in direct contact with the material, were repelled from entering the treated space [17], [18],[27].

As measured by daily RSD, the intra-day variation of airborne DDT in the three-chamber system (<20%) indicate that the replicates are similar and acceptable under EPA testing criteria [25], [28]. Significant differences were observed under laboratory conditions in the concentration of airborne DDT in the treatment chamber measured on different days using treated material newly prepared for each experiment. This difference suggests the amount of DDT that becomes airborne varies by day, although volume and concentration of DDT solution used for material treatment are held constant, which could affect the repeatability of mosquito behavior studies evaluating the same (nominal) treatment conditions. The differences observed are not likely to be a result of sampling or analysis method performance as spiked control samples were within 15% of expected values for laboratory experiments. However, the variation in DDT levels may be due to fluctuations in ambient temperature of the testing room (26–31°C) as it has been shown that the steady state air concentration [20] and vapor pressure [29] of DDT increase in a non-linear fashion at temperatures greater than 28°C. Additional explanations for the inconsistent air concentration of DDT in the treatment chamber include: potential degradation of DDT stock solutions prior to fabric treatment, variations in delivery rate or consistency in the fabric treatment procedure, use of a system that was not air-tight, and sampling under non-equilibrium conditions. However, until correlations can be made regarding thresholds for behavioral responses in mosquito test populations and AI airborne concentrations, the true effect of this variability is unknown. Future studies are planned to investigate the impact of each of these potential confounders on the concentration of airborne DDT within the laboratory.

Although our earlier reports have described air sampling outputs in conjunction with deterrent (SR) mosquito responses [26], this is the first detailed description, to our knowledge, of the conditions and performance of a method for near real-time detection of DDT in samples of air collected under field conditions. The on-site method appeared to be sufficiently sensitive to detect levels well below those that would be acutely toxic to humans (1.0 mg/m^3^) [30] with a quantitation limit of 0.461 µg/m^3^ (27 ppt) DDT during field sampling and analyses. Additionally, the sample collection method developed for this study was relatively simple allowing on-site training of technicians for sample collection. The results of the field analyses indicate DDT was present in the treatment huts and not in the control hut, confirming mosquitoes approaching or entering treatment huts would be exposed to airborne DDT. While the samples collected during this study did not cover each hour of the four-day test period, it could be possible to use the 1 h sampling period to collect consecutive samples to measure variations in the concentration of airborne DDT over time.

A limitation of the method employed for on-site sample analysis was the differences in the method performance, with respect to linearity and intra-sample variability of the controls. These differences may be a result of the operating conditions encountered at the field site (23–32°C; relative humidity 65–100%) compared to those in the laboratory (24–27°C; relative humidity 40–60%). Additionally, strict control of the calibration solutions used during field analysis was not possible, as temperature-controlled shipping options were not used.

The primary strength of this study is the evidence provided that airborne DDT was generated in the laboratory and field test systems used to evaluate mosquito vector behaviors. The data support the conclusion that mosquitoes placed in these systems will be exposed to DDT without landing on treated surfaces. Potential confounders such as material treatment and temperature were identified during these experiments, and these should be controlled or accounted for during future air sampling evaluations. More importantly, the sampling and analysis methods described here validate the role of TD GC-MS in entomological evaluations and overall utility in SR product development. Near real-time analysis can identify operational conditions to optimize for maximum SR product effects. Evaluation of the suitability of TD GC-MS methods for sampling other spatial repellent compounds, such as semi-volatile pyrethroids, as well as other chemical classes that are typically used for vector control is warranted.
